# Orienting Attention Modulates Pain Perception: An ERP Study

**DOI:** 10.1371/journal.pone.0040215

**Published:** 2012-06-29

**Authors:** Sam C. C. Chan, Chetwyn C. H. Chan, Anne S. K. Kwan, Kin-hung Ting, Tak-yi Chui

**Affiliations:** 1 Applied Cognitive Neuroscience Laboratory, Department of Rehabilitation Sciences, The Hong Kong Polytechnic University, Hong Kong, China; 2 Haven of Hope Hospital, Hospital Authority, Hong Kong, China; 3 Department of Anaesthesiology, United Christian Hospital, Hospital Authority, Hong Kong, China; University of Florida, United States of America

## Abstract

**Introduction:**

Research has shown that people with chronic pain have difficulty directing their attention away from pain. A mental strategy that incorporates focused attention and distraction has been found to modulate the perception of pain intensity. That strategy involves placing attention on the nociceptive stimulus felt and shifting attention to a self-generated sub-nociceptive image and rehearsing it. Event-related potential was used to study the possible processes associated with the focus-then-orient strategy.

**Methods:**

Eighteen pain-free participants received different levels of 50-ms nociceptive stimulations elicited by electric shocks at the right lateral malleolus (ankle). In perception trials, participants maintained the perceived nociceptive stimulus in working memory for 3,000 ms. In imagery trials, participants mentally generated and maintained the corresponding sub-nociceptive image they had learned previously. After both types of trials, participants evaluated the pain intensity of the incoming stimulus by recalling the feeling of the nociceptive stimulation at the beginning of the trial.

**Results:**

Shifting attention from the incoming nociceptive to a self-generated sub-nociceptive image elicited central P2 and centro-parietal P3 waves, which were found to correlate with proportional scores on the Stroop Test. They were followed by a frontal N400 and a parietal P600, denoting generation of sub-nociceptive images in working memory. The voltages elicited in these potentials correlated moderately with attenuation of the pain ratings of the recalled nociceptive stimulations.

**Conclusions:**

Focus-and-orient attention across nociceptive and sub-nociceptive images appears to be related to response inhibition. Mental rehearsal of the sub-nociceptive images was found to modulate the perception of the nociceptive sensation felt prior to the imagery. Such modulation seems to be mediated by generating and maintaining sub-nociceptive images in working memory. Future studies should explore the mental processes associated with orienting attention for pain modulation among people with pathological pain and frontal lobe dysfunction.

## Introduction

The somatosensory cortices process afferent nociceptive sensation. Researchers have found that the lateral pathway to the somatosensory cortices mediates feelings of pain intensity, while the medial pathway to the limbic system mediates the affect resulting from that pain [1], [2]. When people down-regulate the sensation of pain, the central nervous system exerts efferent inhibitory control. This control is mediated by the periaqueductal grey, situated in the midbrain. Wiech and colleagues [3] found that conscious down-regulation was associated with activity in the so-called “pain control center” located in the dorsolateral prefrontal cortex.

Common down-regulation strategies that help lessen pain include distraction [4]–[8] and focused attention [9]–[11]. Distraction involves reorienting attention from a pain sensation to a sensation that occurs at the same time, but is not the pain itself (a sensation or scenario) [12]. The common modalities used to initiate distraction are visual [17]–[18], auditory [20], or somatosensory [5], [6], [21]. Studies have still not given a clear picture of exactly what mental processes people use during distraction from nociceptive sensation [10], [13], [14].

Evidence from studies on distraction suggests that it is hard to actually shift attention away from pain using distraction [3], [15]. A fundamental problem in mental distraction is that participants need to focus their attention on attributes that are not a part of the painful stimulus [16]. In a review paper, van Damme and colleagues [16] concluded that non-painful stimuli were less useful than pain-related stimuli for down-regulating bodily pain. They explained that participants are less motivated when the content of the intervention is not directly related to pain sensation. Other researchers suggested that the effect of distraction on modulating pain perception could be compromised by hypervigilance or failure in attentional control on pain sensation developed among participants with chronic pain [12], [22].

Another strategy for modulating pain perception is focused attention. Unlike distraction, focused attention has people attend to the sensory component of the nociceptive sensation, such as the intensity or location of the sensation (e.g., [9], [10]). Previous studies have found that focused attention is effective at modulating pain sensation among healthy participants [11] and people with chronic pain [9], [10]. The parallel treatment model proposed by Leventhal and colleagues offers an explanation the effect of focused attention [23]–[25]. This model proposes two exclusive parallel systems in pain networks that carry discriminative and emotional nociceptive information [26]. The lateral system constitutes the cognitive aspect of nociceptive signals (such as location and intensity in the somatosensory cortices), and the medial system carries affective signals to the emotional systems. Under this architecture, one could alter pain perception by separating the objective schema from the subjective counterpart. The focused attention strategy would thus enable pain modulation by focusing on the objective representation of pain (such as intensity) and setting aside the subjective representation (such as anxiety) [9]–[11].

Nouwen and colleagues [10] compared the effects of focused attention and distraction on cold-induced pain in people with lower back pain and healthy controls. In the focused attention condition, participants were asked to continually verbalize the sensation they perceived during a 7-minute exposure to cold pain. For both groups, focused attention was more effective than distraction at attenuating pain. Moseley and colleagues [9] used a more sophisticated focused attention design that had patients with complex region pain syndrome discriminate tactile stimuli applied to the back of the hand. When the patients felt the cork probes, they had to identify the diameter of the stimulus and where it was applied. The discrimination task attenuated the patients’ pain. The authors explained that pain modulation because the task directed the patients’ attention away from the chronic pain to the physical characteristics of non-pain-related (sub-nociceptive) parts of the tactile stimulus.

Several studies have explored the mechanism underlying focused attention and nociceptive perception [5], [27], [28]. Hatem and colleagues [5] found that Nogo-N2 (150–400 ms) and Nogo-P3 (300–500 ms) were elicited by a laser, but not electricity-induced pain. These two components originate from the fronto-central regions of the scalp and were found to be functionally related to inhibitory and response conflicts [5]. Using a cue-validity paradigm, Dowman [27], [29] found two different ERP components related to pain modulation. More positive-going P2 and P3a at central sites were elicited when participants were presented with visual cues before perceiving electrical nociceptive stimuli. The visual cues were meant to further orient participants’ attention away from the evoked stimuli. Dowman further argued that the central P2 is related to spatial shifting of attention to the evoked stimuli, whereas the anterior P3a reflects processing involving attention and working memory [27], [30]. Their findings revealed the mental processes that possibly underlie regulation of pain perception using the focused-attention strategy.

Focused attention has been regarded as a better strategy than distraction for pain modulation because it motivates people to directly address the pain sensation. Nevertheless, a few studies have reported that participants failed to continue the procedure because directing attention on the objective component could further intensify the pain sensation [9], [10]. This was particularly the case for people with severe pain. Even though it seems to work, the idea that directing focus onto the objective component will attenuate pain is still counter-intuitive.

This study attempts to combine the advantages of the focused-attention and distraction strategies for pain modulation, what we call “orienting attention.” In our design, participants first feel a brief nociceptive stimulus at the beginning of the trial and then bring to mind the image of the nociceptive stimulus or a corresponding sub-nociceptive image. Then they rate the pain they felt at the beginning of the trial. The focused-attention component is meant to place attention on the nociceptive stimulus felt, whereas the distraction component is meant to switch attention to a self-generated sub-nociceptive image and rehearse it before rating the pain.

We intended to explore the neural processes that occur when people orientate their attention from nociceptive to sub-nociceptive images while regulating nociceptive perception. This study required participants to orient attention among nociceptive and sub-nociceptive experiences, as well as mentally rehearse well-learned nociceptive and sub-nociceptive images. The design was meant to improve the signal-to-noise ratio of the electrophysiological signals in two ways. First, we used a 50 ms nociceptive stimulus, which would evoke relatively large potentials (>10 mV) [29], [35] that would be less likely to interfere with the weaker signals associated with the imagery processes (<5 mV) [31]–[33] occurring later in the trials. Second, ensuring that participants have learned to generate and rehearse the images well can further reduce the variability of the signals.

We anticipated that the early neural processes elicited from placing attention on the brief nociceptive stimulus would be similar to the findings of some behavioral and neurophysiological studies on focused attention. For example, in Nouwen’s behavioral study [10], participants with chronic lower back pain were exposed to cold pressor pain for 7 minutes. In the focused-attention condition, they attended to the nociceptive sites and verbally described the nature and characteristics of the sensation. Increased amplitude of P2 at the central region was elicited in response to the attention placed on the nociceptive stimulations applied to the participants’ wrist [35]. Researchers regard the P2 component as an index of cognitive processing of pain-related endogenous parameters, including spatial localization [34], [35]. Another study found that more positive-going amplitude was also associated with the spatial shifting of attention to the evoked stimuli [30].

Research has found that the P300 component is associated with evaluation of the stimulus intensity attended to [30], so it might follow the P2. We hypothesized that cueing the shift from the nociceptive to sub-nociceptive image would elicit reorientation of attention, denoted by fronto-central P2 and an anterior P3 after that. Attention reorientation should also involve inhibitory responses because it requires reorienting attention to modalities other than the nociceptive stimulus. The mental rehearsal of the sub-nociceptive image would involve access to working memory denoted by a frontal N400 and maintenance of the somatosensory image denoted by a parietal P600 [28], [30]. These components should be related to the behavioral results–i.e., the amount of change in the pain scale due to the rehearsal of the self-generated sub-nociceptive images [10].

This study makes several contributions. This study can extend our understanding of how focused attention followed by distraction (generating and rehearsing sub-nociceptive images) modulates nociceptive experiences. It can also shed light on the potential of using self-generated sub-nociceptive sensations to develop clinical interventions for pain modulation for people with chronic pain.

## Methods

### Ethics Statement

The research committee of the Department of Rehabilitation Sciences of The Hong Kong Polytechnic University approved this study. The ethics committee of the same department approved the experimental procedure and written consent form. All participants gave written informed consent.

### Participants

Eighteen healthy volunteers (11 female) free of neurological deficits and psychiatric disorders were recruited via convenience sampling. Participants were recruited through advertisements placed on notice boards around the university campus. Potential participants who were interested in the study contacted the investigator (SCCC) by phone or e-mail for details. An initial appointment was set up to explain the purpose of the study, obtain informed consent, and screen for inclusion criteria. All of the participants were right-handed. Mean age was 35.8 years (SD = 13.2 years).

### Somatosensory Stimuli

The somatosensory stimuli were generated from the Grass S48 stimulator connected in series with a Grass CCU1 constant current unit (Grass-telefactor, West Warwick, RI). The somatosensory stimuli consisted of a 25-pulse train of electrical square-wave pulses (0.5-millisecond pulse duration and 500 Hz frequency), which was similar the procedure of Katayama and colleagues [34]. The train duration was set to 50 ms. A constant current unit regulated the output from the stimulator to ensure the current was stable for each pulse train. During the experiment, the CCU1 unit adjusted the current intensity.

A total of 10 individually calibrated stimuli were constructed for each of the participants, with five sub-nociceptive stimuli and five nociceptive stimuli. They were constructed by obtaining the painful and sub-painful thresholds of each participant with the gradual stepping-up and stepping-down method described by De Pascalis and colleagues [35]. The first threshold was the minimal detectible sensation. To get the minimal detectible sensations, participants received a series of ascending single pulse trains that lasted 50 ms from 0.0 mA in increasing increments of 1 mA until participants reported feeling a sensation. The descending counterpart of the procedure was to start at 1 mA above the minimal detectible sensation and decreased in steps of 1 mA until participants reported not feeling the sensation. The two thresholds obtained from the ascending and descending procedures were then averaged to determine the averaged minimal detectible sensation.

The second threshold was the sub-pain threshold. To get the sub-pain threshold, participants received increasing electrical stimulations in steps of 1 mA until they reported feeling a minimal pinprick sensation. Then, as before, the sub-pain threshold was tested using descending shocks. The ascending and descending pain thresholds were then averaged to obtain the average sub-pain threshold. Participants were told to stake this level of pain as “1” on the pain numerical rating scale (NRS). The range of voltage intensity between the minimal detectable sensation and the sub-pain threshold formed the sub-painful sensation range.

The third threshold was the “very painful” sensation. To get the very painful sensation, participants received increasing electrical stimulations in steps of 1 mA above the sub-pain threshold. After the stimulation, participants rated the intensity of the pain on the 11-point NRS. The ascending procedure continued until participants gave a pain NRS of 7, which was labeled as a “very painful” sensation. The range of voltage intensity between the sub-pain threshold and “very painful” sensation formed the painful sensation range.

The mean voltage for the minimal detectible sensation was 3.50 mA (SD = 2.92 mA); the mean sub-pain threshold (NRS = 1) was 12.10 mA (SD = 9.10 mA); and the “very painful” sensation (NRS = 7) was 22.80 mA (SD = 12.93 mA). The five sub-nociceptive stimuli were derived by evenly distributing them along the sub-painful sensation range of each participant (i.e., between the minimal detectible sensation and the sub-pain threshold), e.g., 3.50–12.10 mA/6. The five nociceptive stimuli followed the same procedure along the painful sensation range (i.e., between the sup-pain threshold and “very painful” sensation), e.g., 12.10–22.80 mA/6.

After the calibration and before the experiment, each participant received training on differentiating the stimuli. There were three parts in the training. First, the participant was trained to differentiate the sub-nociceptive stimuli in an ascending order. After the delivery of one stimulus (25-pulse train lasting 50 ms), participants were instructed to attend to the stimulus and remember the sensation it generated. There were two presentations for each stimulus. The participants repeated the same procedure for each of the five sub-nociceptive stimuli at their own pace.

After the learning phase, the sub-nociceptive stimuli were delivered to the participant in a random order. Participants identified each stimulus by specifying the level of the stimulus from 1 (weakest level) to 5 (strongest level). The experimenter gave verbal feedback on the accuracy of the responses. The initial number of training trials was 100, with 20 for each level arranged in a pseudo-randomized order. The required accuracy rate was set at 80%. Participants completed additional trials if they failed to achieve the pre-determined competence level (only a few participants [<10] required additional trials). The same learning and testing procedures were conducted for participants to get familiar with the other five calibrated nociceptive stimuli (from 1 to 5).

Next, participants were trained to pair up the corresponding levels of sub-nociceptive and nociceptive stimuli–for example, level 1 of the sub-nociceptive stimulus corresponds to level 1 of the nociceptive stimulus. For this training, one level of nociceptive stimulus was given, followed by another level of sub-nociceptive stimulus, which could be at the same or different level. Participants had to tell whether the levels of the two nociceptive and sub-nociceptive stimuli matched or not. The initial number of training trials was 50, and the competence level was again set at 80% accuracy. Additional trials were given for those who could not reach the competence level in the first round (only a few [<10] required additional trials).

Third, since the perception or imagery trials were presented in a random order, participants were required to learn to associate two auditory cues with the designated mental processes. A low-pitched cue (500 Hz) indicated that they were to perceive the nociceptive stimulus; a high-pitched cue (1,500 Hz) indicated that they were to generate and rehearse the sub-nociceptive stimulus. There were 20 trials for the participants to achieve 80% accuracy (no additional trials were needed). None of the training involved rating the intensity, except for labeling the levels of the stimuli from 1 to 5. This should minimize potential interference between the subjective rating of pain intensity and the 11-point NRS in the task.

### Task Paradigm

**Figure 1:**
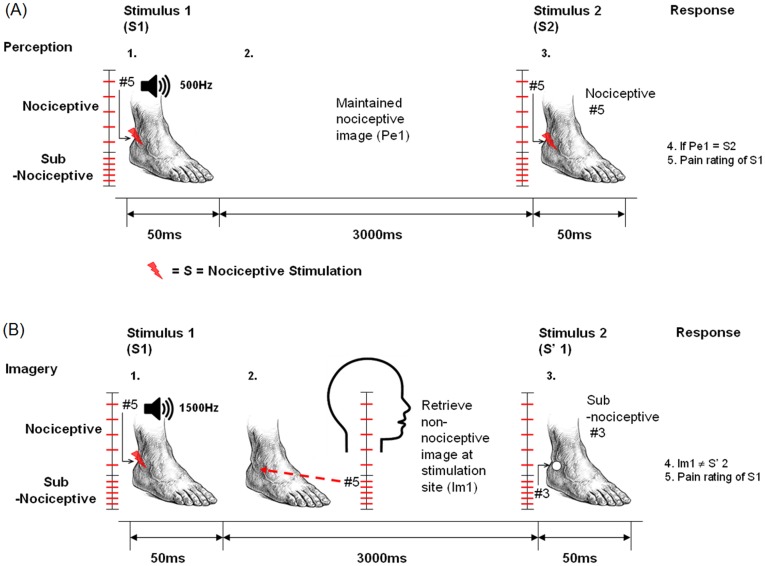
Diagrammatical representation of the experimental paradigm. (A) Perception trial Note: 1. A nociceptive stimulus #5 (S1) was delivered to the participant’s lateral malleolus coupled with a low-pitched tone which both lasted for 50 ms. 2. The participant perceived the stimulus for 3,000 ms and maintained the image. 3. A nociceptive stimulus #5 (S2) was delivered to the site for 50 ms; 4. The participant was to respond by stating whether S2 would have been at the same intensity level to that of the nociceptive image maintained during the 3000 ms (Pe1); the participant should respond “yes.” 5. The participant rated the nociceptive image from S1 on an 11-point NRS. (B) Imagery trial Note: 1. A nociceptive stimulus #5 (S1) was delivered to the participant’s lateral malleolus coupled with a high-pitched tone, which both lasted for 50 ms. 2. The participant generated a sub-nociceptive image #5 (Im1) and mentally rehearsed the sub-nociceptive image for 3,000 ms. 3. A sub-nociceptive stimulus #3 (S’1) was delivered to the site for 50 ms. 4. The participant was to respond by stating whether S’1 would have been at the same intensity level to that of Im1; the participant should respond “no.” 5. The participant rated the nociceptive image from S1 on an 11-point NRS.

In the experiment, participants completed both perception and imagery trials (Figure 1). In perception trials, participants heard a low-pitched tone (500 Hz, 60 db) to signify a perception trial. At the same time, they felt a 50-ms electric shock of intensity (called S1) from one of the five calibrated nociceptive sensory stimuli on their lateral malleolus area (near the outer side of the ankle). Participants were told to maintain the nociceptive image for 3,000 ms before receiving a second nociceptive stimulus (called S2). They had to determine whether the maintained image (Pe1) and S2 were at the same level. At the end of each trial, participants had to recall and rate the perception of the recalled nociceptive image from S1 using the 11-point NRS [37].

In imagery trials, the schedule of presenting the 50-ms nociceptive stimulus (S1) was the same as in the perception trials. But instead of a low-pitched tone, participants heard a high-pitched tone (1,500 Hz, 60 db). In the 3,000 ms period, participants had to generate and maintain the feeling of the sub-nociceptive image at the corresponding intensity level of S1 (called Im1). This required participants to recognize the level of S1 (such as level 5) and then access the memory of the corresponding sub-nociceptive somatosensory characteristics (also 5) they had learned during the training session. Participants then received a sub-nociceptive stimulus (S’1). After receiving S’1, participants had to decide whether Im1 (an imagined sensation) and S’1 (a felt sensation) were at the same level. As in perception trials, they recalled and rated the perception of the nociceptive image from S1 using the 11-point NRS.

All five levels of electrical stimulations for the first and second stimuli and the pairing of the auditory cue and the first stimulus were pseudo-randomized. The ratio of high- and low-pitched auditory cues was 1∶1. In half of the trials, the stimuli for comparison were at the same level. The intensive training and high mastery level (above 80%) participants attained ensured that they could pair the nociceptive stimuli with the corresponding sub-nociceptive stimuli during the experiment.

There were 20 trials in each block, with 10 pseudo-randomized perception and 10 pseudo-randomized imagery trials. Each participant received eight blocks that were randomly selected from 16 pre-determined blocks. This gave a total of 160 trials (80 perception and 80 imagery trials). Each trial lasted 3,100 ms. The interstimulus interval was at least 10 s. Thus each block lasted for about 262 s. Participants were offered long breaks to avoid mental fatigue and overstimulating the skin.

### Procedure

After the training, participants first completed a demographic questionnaire and the Stroop Test (administered by the experimenter) in a distraction-free corner of the laboratory. Participants then started the experimental paradigm by sitting in a comfortable chair in front of a table with their arms and back well supported. They placed both feet comfortably on the floor with their thighs positioned horizontally to the floor. A footstool and a back cushion were used to provide stable support whenever needed. The computer monitor that presented visual stimuli (such as instructions about the blocks) to participants was placed 60 cm away from the participants. Two speakers were placed on either side of the monitor to deliver biaurical pitches. The positive and negative Ag/AgCl electrodes (8 mm in diameter) that gave the shocks were filled with electro-conductive hypocollagen gel.

The positive electrode was secured at the right lateral malleolus, which is sensed by the sural nerve (L5-S1 dermatome) [27], [29], [38], [39]. The right lateral malleolus was selected because it will be relevant for future studies on diagnostic groups, such as people with lower back pain. Electrical stimuli parameters were based on specifications from Katayama and colleagues’ study [36], which used a 25-pulse train of stimuli (0.5 ms pulse duration and 500 Hz frequency).

#### Stroop Test

Studies have shown that the frontal lobe mediates pain modulation [3], so participants took the Stroop Test as a measure of their ability to monitor and resolve conflict [40], [41]. This study used the Chinese version of the test [31]. The test has three parts: word reading (WR), color naming (CN), and incongruent color naming (INC).

In the WR block, participants took 100 trials on which they saw a white background with a Chinese color name was printed in black (“
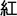
” [red], “
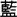
” [blue], “
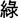
” [green], and “
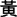
” [yellow]). These words were organized randomly on a 10×10 array. Participants had to read each word out loud as quickly and accurately as possible.

In the CN block, participants saw another background on which the same color words were printed. Unlike the previous block, the words were printed in congruent colors (for example, “red” printed in red). Participants read each word as fast and accurately as possible.

In the INC block, the words were printed in incongruent colors (for example, “red” printed in blue, green, or yellow) and presented to participants on a different background. Participants named the color of each word (not read the word) as fast and accurately as possible. The researcher recorded the number of mistakes made by the participant and the number of self-corrected mistakes in each part of the test. The experimenter recorded the time the participant took to read the words with a digital timer. The difference and proportional scores were computed using the same method as previous studies [40], [41].

### EEG Recording Parameters

Event-related potentials (ERP) recording took place in the soundproof chamber of the Applied Cognitive Neuroscience Laboratory in The Hong Kong Polytechnic University. The ERP signals were captured by a NuAmps Digital DC EEG Amplifier with 128 channels using 90mm Ag/AgCl sintered electrodes (NeuroScan Inc., Sterling, VA). Vertical and horizontal electrooculograms (EOGs) were recorded by two pairs of electrodes to monitor eye movements and blinks. The EEG signals were amplified and digitized at a sampling rate of 1,024 Hz. The montage was referenced to the left and right mastoid processes, and the ground electrode was placed on the forehead in front of the vertex electrode (Cz). The 128-channel Quikcap was connected to the two head-boxes of the SynAmps2 Digital DC EEG Amplifier. The configuration of the electrode positions was pre-defined according to the SynAmps2 Digital. Reference impedances were set to less than 5 kΩ. The timing and presentation of all the output stimuli were coordinated with the synchronization stimulus presentation program STIM2 (NeuroScan Labs, Sterling, VA).

Only those trials in which the participants correctly matched the maintained nociceptive image (Pe1) and S2 in perception trials and the rehearsed sub-nociceptive image and S’1 in imagery trials were selected for subsequent analysis. During preprocessing, the electrophysiological data acquired at all channels was re-referenced to the average mastoid reference converted from the left and right online mastoid reference electrodes. Ocular artifact reduction was applied to the re-referenced data using a regression algorithm in NeuroScan 4.3. Sections of EEG data ranging from 100 ms pre-stimulus to 1,000 ms post-stimulus were epoched, followed by baseline correction against the pre-stimulus interval. Epochs with amplitudes larger than 100 mV were rejected. The remaining epochs were then averaged among the perception and imagery trials. The grand averaged signals were then digitally filtered with a “Zero Phase Shift” filter with a low-pass of 30 Hz and 24 db/oct.

### Data Analysis

The analysis only included correctly matched trials in perception and imagery trials. The mean NRS on the recalled nociceptive images were computed for all levels of nociceptive stimuli in each of the two task trials. A two-way repeated-measures ANOVA was used to test the effects of trial (perception and imagery) and pain level (levels 1 to 5). The significance levels of post-hoc tests across the five pain levels were adjusted using Bonferroni’s corrections (corrected *p* = .01). The mean response time, mean accuracy, and total number of self-corrections were obtained for each of the WR, CN, and INC blocks for the Stroop Test. The difference and proportional scores based on response time were computed.

The standardized ERP epoch grand average and componential analysis were computed with NeuroScan 4.3 (NeuroScan Inc., 2009). In order to extract the appropriate time window for componential analysis, independent component analysis was conducted using EEGLAB software with MATLAB 11.0. This was particularly useful for components with close temporal proximity, such as P2 and P3. The filtered EEG signals from 128 channels collected from the imagery trials were decomposed into the same number of independent components (IC). Each IC had a distinctive scalp distribution and a specific activity course, which designated the onset and offset of that particular IC. We selected the ICs that had clear spatial topography, contributed the most energy (in µV), and showed time courses consistent with the hypothesized neural processes in the experiment for subsequent conventional componential analysis [42]. Because preliminary analysis indicated that amplitudes of the ERP components were independent of the levels of electrical stimulation, epochs were pooled in the subsequent analyses. This pooling increases the power of the analyses.

To test for the effect of condition, the average baseline-to-peak amplitudes of each chosen component were submitted to a two-way repeated-measures ANOVA: 2 (perceptual and imagery trial) ×6 midline sites (Fz, FCz, Cz, CPz, Pz, and POz) [27], [33]. To test for the effect of site, we computed an additional three-way repeated-measures ANOVA: 2 conditions × laterality ×7 lateral sites on either hemisphere (F3, FC3, C3, CP3, P3, PO3, and T7 on the left and F4, FC4, C4, CP3, P4, PO4, and T8 on the right). A similar two-way repeated-measures ANOVA for midline electrode sites and a three-way repeated-measures ANOVA for lateral electrode sites were used to compare the differences in component latency. Greenhouse-Geisser corrections were used. The uncorrected degrees of freedom and the value of epsilon (ε) were used to adjust the significance level of the ANOVAs. Post-hoc tests were conducted using Bonferroni’s corrections (corrected *p* = .05/7 = .01).

To explore the extent to which pain modulation might be subserved by prefrontal lobe function, Pearson’s correlations were computed between the pain NRS on pain intensity (separate for perception trials: NRS_Perception_ and imagery trials: NRS_Imagery_), amplitudes of individual ERP components, and subtest scores on the Stroop Test. The procedures were repeated using the normalized pain NRS on the recalled nociceptive image (NRS_Imagery_ – NRS_Perception_) instead of the raw pain NRS. The normalized pain NRS were calculated by subtracting the pain NRS on the recalled nociceptive images in the imagery trials from those in the perceptual trials. In other words, normalized pain NRS is meant to reflect the extent of the pain modulation due to rehearsing the sub-nociceptive image in relation to the recalled nociceptive image.

## Results

### Behavioral Data

The mean NRS of the recalled nociceptive images for the five levels of nociceptive stimuli during perception trials ranged from 2.51 (SD = 1.51, for level 1) to 4.87 (SD = 1.76, for level 5; see Table 1). During imagery trials, the mean ratings ranged from 2.17 (SD = 1.44, for level 1) to 4.83 (SD = 1.54, for level 5). The average pain NRS across the five levels for perception and imagery trials was 3.97 (SD = 1.81) and 3.68 (SD = 1.75) respectively. The average normalized NRS was -0.33 (SD = 0.44), which suggested a decrease in ratings in the imagery trials.

There were significant effects of condition *F*(1, 17) = 10.67, *p*<.005 and pain level *F*(1, 24) = 34.82, *p*<.001. The post-hoc analyses suggest that the perceived pain intensities for levels 1 to 3 in the imagery condition were significantly lower than those in the perception condition *t*(17) = 2.63 to 3.52, *p*s <.01. The between-condition differences in the NRS on the recalled levels of 4 and 5 nociceptive images were not statistically significant.

**Table 1:** Numerical rating scale (NRS) ratings of 1 to 5 level nociceptive images in Imagery and Perception conditions.

	Levels of Nociceptive Sensation					
	Level 1	Level 2	Level 3	Level 4	Level 5	Average
Imagery	2.17 (1.44)	2.77 (1.60)	3.43 (1.59)	4.07 (1.54)	4.83 (1.54)	3.68 (1.75)
Perception	2.51 (1.51)	3.10 (1.60)	3.79 (1.70)	4.41 (1.73)	4.87 (1.76)	3.97 (1.81)
I – P	−0.34 (0.38)	−0.34 (0.40)	−0.35 (0.52)	−0.34 (0.72)	−0.04 (0.45)	−0.33 (0.44)

Note: I – P  =  Differences in NRS ratings between the Imagery and Perception conditions. Standard deviations are in parentheses.

Table 2 summarizes the results of the Stroop Test. The scores on the Stroop Test correlated most highly with level 4 normalized pain NRS, followed by level 1 and average normalized pain NRS (Table 3). Among the different scores, the proportional scores of the Stroop Test correlated moderately with level 1, 3, 4 and average normalized pain NRS. Among them, the (INC-WR)/WR and (INC-CN)/CN yielded the highest proportional scores with level 4 normalized pain NRS (*r*s = .632 and .679, *p*<.01, respectively). Their relationships with the average normalized pain NRS were less strong (*r* = .486 and .425, *p*<.05, respectively).

**Table 2:** Participants’ scores on the Chinese version Stroop Test.

	Mean (SD)
Mean response time (sec) (SD)	
WR	49.28 (12.81)
CN	73.12 (21.29)
INC	121.65 (29.31)
Difference scores (sec) (SD)	
CN-WR	23.83 (16.31)
INC-CN	48.52 (21.08)
CN-WR	72.36 (21.16)
Proportional scores (SD)	
(CN-WR)/WR	0.51 (0.33)
(INC-CN)/CN	0.70 (0.27)
(INC-WR)/WR	1.51 (0.43)

Note: WR = Word reading; CN = Color Naming; INC = Incongruent color naming; SD = standard deviation. Different scores are computed by subtracting the reaction time score of the earlier from the later test. Proportional scores are computed by dividing the difference scores by the total time of the earlier test.

**Table 3:** Correlations between the normalized pain NRS on different levels of nociceptive images and the Stroop Test scores (included only those with p<0.05).

	Normalized Pain NRS			
Stroop Test	Level 1	Level 3	Level 4	Average
*Raw Scores*				
WR Time	–0.546*			
WR Error			–0.492*	
CN Error	0.607**	0.526*		0.443*
*Difference Score*				
INC–WR			0.481*	
INC–CN			0.498*	
*Proportional Scores*				
(INC–WR)/WR	0.578*		0.632**	0.486*
(INC–CN)/CN		0.545*	0.679**	0.425*

Note: Normalized Pain NRS = NRS_Imagery_–NRS_Perception_. Average normalized pain NRS is computed by averaging the normalized pain NRS across five levels of stimulation.

Key: WR = Word reading; CN = Color Naming; INC = Incongruent color naming. Different scores are computed by subtracting the reaction time score of the earlier from the later test. Proportional scores are computed by dividing the difference scores by the total time of the earlier test.

* p<0.05.

** p<0.01.

No significant correlations were obtained for level 2 normalized pain NRS (mostly r <0.40). Only one significant correlation was obtained for level 5 normalized pain NRS with CN Time Error (p = 0.566, p<0.05).

### Electrophysiological Data

The percentage of correct trials ranged from 41.7% (SD = 6.8%; level 1) to 67.6% (SD = 19.7%; level 5). The average number of trials submitted for analysis on the perception trials ranged from 6.2 (SD = 1.5) for level 1 to 8.7 (SD = 1.7) for level 5. On imagery trials, it ranged from 5.8 (SD = 1.8) for level 1 to 8.8 (SD = 1.5) for level 5. The number of artifacts identified was generally similar across the two conditions and different stimulation intensities.

**Figure 2:**
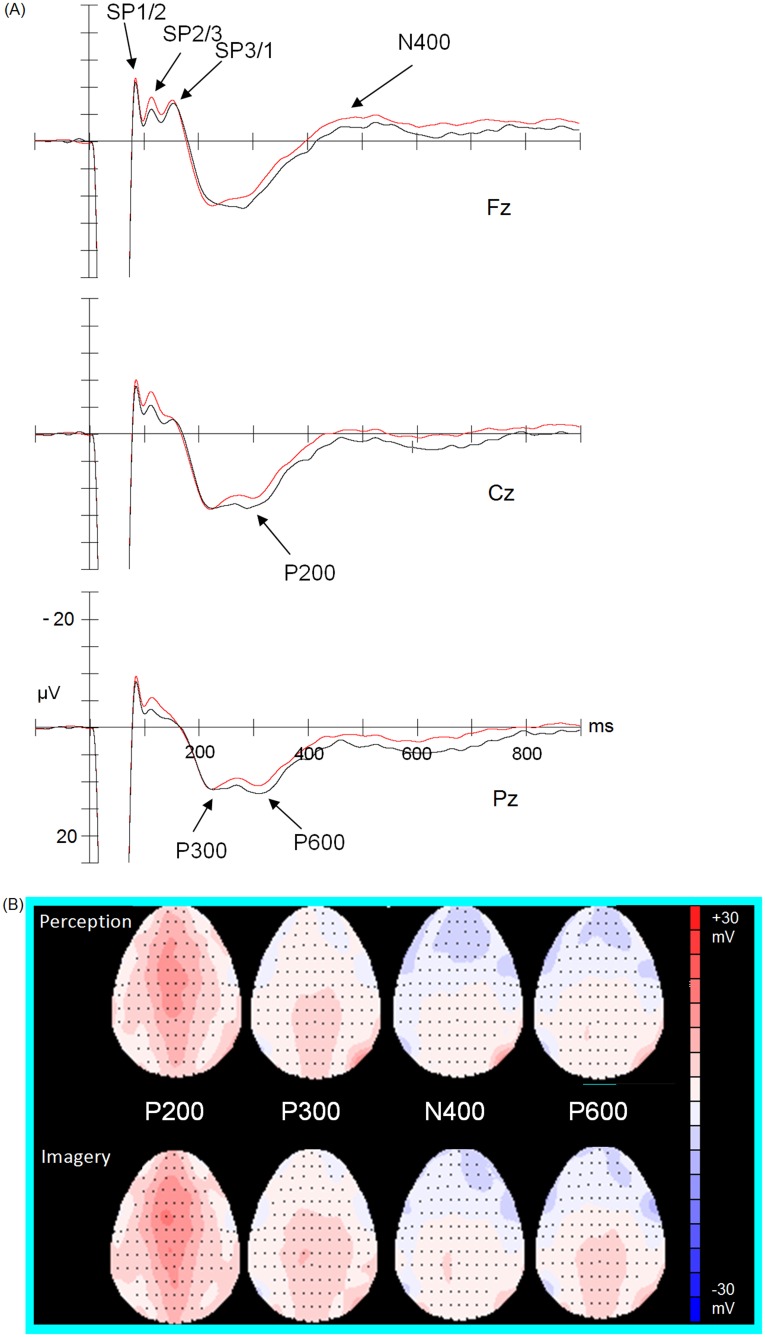
Event-related potentials and their topography captured during the task. (A) Grand average event-related potentials of imagery (black) and perception (red) at three midline sites. The amplitudes of the positive potentials (not shown in figure) peaked around 32 ms are 113.5 mV for Fz, 101.1 mV for Cz and 92.6 mV for Pz. This could be due to the artifact generated by the electrical current emitted from the stimulator. (B) Grand average of four ERP topographic patterns of imagery and perception conditions.

The following independent components were identified: stable period (SP) 1–2 (73–101 ms), SP2–3 (101–129 ms), SP3/P1 (133–173 ms), P1 (177–265 ms), P2 (273–341 ms), P3 (349–409 ms), N400 (411–475 ms), and P600 (known as the later positive component or LPC; 507–650 ms; see Figure 2). There were significant effects of condition on amplitudes in the SP1/2 (condition: *F* [1], [17] = 14.01, *p*<.01; midline site effect: *F*[5, 85] = 2.66, *p*>.05, ε = 0.43; condition × midline site: *F*[5, 85] = 1.03, *p*>.05, ε = 0.41), and SP2/3 (condition: *F* [1], [17] = 13.33, *p*<.01; midline site effect: *F*[5, 85] = 11.28, *p*<.05, ε = 0.33; condition × midline sites: *F*[5, 85] = 0.42, *p*>.05, ε = 0.40).

The lateral site effects were all non-significant. For the SP1/2, amplitudes during imagery trials were significantly less negative than during perception trials. SP1/2 amplitudes peaked at Fz (imagery: −11.08±6.02 µV; perception: −12.15±5.52 µV) and then at Cz and FCz (*p*<.001; Figure 1a). SP2/3 was less negative in the imagery trials than in the perception trials, where it peaked at FCz (imagery: −7.31±5.99****µV; perception: −9.85±5.59****µV) and then at Fz and Cz (*p*<.001).

For SP3/P1, the mean amplitudes peaked at Fz in both the imagery (−8.87±4.65 µV) and perception (−9.52±4.57 µV) trials. The effect of site was significant (midline site: *F*[5, 85] = 12.88, *p*<.005, ε = 0.41; condition × midline site: *F*[5, 85] = 1.81, *p*>.05, ε = 0.35), but the effect of task was not significant (*F* [1], [17] = 0.01, *p*>.05). The lateral sites effects were not significant (condition × laterality: *F* [1], [17] = 1.33, *p*>.05; condition × laterality × site: *F*[6, 102] = 0.76, *p*>.05, ε = 0.46; Figure 2). The P1 component peaked at CPz (imagery: 16.88±7.05****µV; perception: 16.42±7.97****µV) during both types of trials. The midline site effect was significant, suggesting a bilateral frontal distribution, but the condition and interaction effects were not significant (condition: *F* [1], [17] = 0.13, *p*>.05; midline site: *F*[2.58, 43.89] = 13.81, *p*<.005; condition × midline site: *F*[5, 85] = 0.87, *p*>.05, ε = 0.26). The laterality effects and its interaction effects were all not significant.

The P2 component had similar topographical distributions as the P1. The mean amplitudes also peaked at CPz during imagery (17.17±6.97****µV) and perception trials (14.80±7.63 µV). There was a significant midline site effect (condition: *F* [1], [17] = 12.24, *p*<.01; condition × midline site: *F* [5], [58] = 1.89, *p*>.05, ε = 0.25), but no laterality sites effects were significant. Waves during imagery trials were more positive than during perception trials at FCz and Cz (*p*<.001). The P3 component peaked at PCz during both imagery (10.83±4.93****µV) and perception trials (8.74±5.94****µV). There were significant differences in the midline site effect between the two task conditions (condition: F[1], [17] = 9.02, *p*<.01; condition × midline site: F[5, 85] = 0.950, *p*>.05, ε = 0.32) but not among any of the lateral sites. The imagery trials were more positive-going than the perception trials, with the largest differences at FCz and Cz (*p*<.01).

The N400 component peaked at Fz in imagery (−3.84±6.70 µV) and perception trials (−5.30±6.39 µV). The effect of midline site was significant (condition: *F* [1], [17] = 9.21; *p*<.01; condition × midline site: *F*[5, 85] = 1.21; *p*>.05, ε = 0.32), with the waves on imagery trials less negative than on perception trials at FCz (*p*<.005). There were no significant laterality site effects (condition × laterality: *F* [1], [17] = 0.01, *p*>.05; condition × laterality × site: *F*[6, 102] = 0.27, *p*>.05, ε = 0.45).

The P600 peaks were distributed more toward the posterior at CPz (imagery: 6.71±4.25 µV; perception: 3.92±4.23 µV). The midline site effect was also significant (condition: *F* [1], [17] = 10.37; *p*<.01; condition × midline site: *F*[5, 85] = 1.15, *p*>.05, ε = 0.42), with waves on the imagery trials more positive than on perception trials in all sites, except Fz (*p*<.01). The analyses for laterality effects were all non-significant. Unlike with amplitudes, none of the comparisons of the latency of the ERP components was statistically significant.

In sum, the condition effect was significant on the amplitudes of SP1/2 and SP2/3, with the imagery trials less negative-going than the perceptual trials at the frontal and central regions. There were no significant findings for SP3/P1 and P1. For the late components (P2 to P600), there were significant midline site effects on amplitude, with the imagery trials more positive-going than the perceptual trials over extensive areas from frontal to parietal regions (or less negative-going for N400).

### Magnitude of ERP Components and Pain Modulation

This section reports the results based on normalized pain NRS ratings. Correlations based on raw pain NRS were not significant. It is noteworthy that most of the significant correlations were between P2, P3, and N400 components and level 1 and 5 normalized pain NRS, followed by the average normalized pain NRS (Table 4). The level 1 normalized rating correlated consistently and moderately with the fronto-centrally distributed P2, P3, and N400 components. In contrast, level 5 normalized ratings consistently correlated with left frontally distributed P2, P3, and N400. There was the same pattern of correlations (frontally oriented) for the average normalized pain NRS. P2 waves correlated most extensively with levels 1, 3, and 5, as well as with the average normalized ratings. The patterns of the P3 and N400 waves were reversed: P3 correlated most with level 1, and N400 correlated most with level 5. The P6 in general correlated less with the normalized pain NRS.

**Table 4:** Correlations between the normalized pain NRS on different levels of nociceptive images and the amplitudes of the later ERP components at selected sites (included only those with p<0.05).

	Normalized Pain NRS			
	Level 1	Level 3	Level 5	Average
*P2 Component*				
F4	–0.501	–0.549	–0.469	–0.456
Cz	–0.455			
P3	–0.444	–0.441		
P3 *Component*				
F4	–0.556		–0.483	–0.436
Cz	–0.582			
P3	–0.497		–0.449	–0.414
*N400 Component*				
F4	–0.470		–0.549	–0.418
Cz	–0.530		–0.417	–0.406
P3			–0.524	
P600 *Component*				
F4			–0.482	
Cz	–0.489			

Note: All significant level was p<0.05; Normalized Pain NRS = NRS_Imagery_–NRS_Perception_. Average normalized pain NRS is computed by averaging the ratings across five levels of stimulation.

No significant correlations were obtained for levels 2 and 4 normalized pain NRS.

## Discussion

### Key Findings

This study investigated the neural processes behind orienting attention from nociceptive to sub-nociceptive images in order to regulate nociceptive perception. This focus-then-orient attention approach shares the benefits of focused attention and distraction (or orienting) for modulating pain perception. The “focusing” component involved attending to the nociceptive stimulus and recognizing its intensity, while the “orienting” component involved generating and rehearsing a corresponding sub-nociceptive image from memory. Behaviorally, participants might feel less pain from the recalled nociceptive images after they mentally rehearsed the sub-nociceptive images (imagery trials). However, the differences in the presentation schedule for the stimulus verification process (two times for perception trials versus one time for imagery trials) between the two conditions might confound the observable pain modulation effects.

P2 amplitudes–which are thought of as “markers” of attention shifts–were more positive during imagery than perception trials, peaking at FCz and Cz. Differences were also found in the components that reflect generation and maintenance of self-generated sub-nociceptive images in working memory: P3, N400, and P600. Amplitudes in these regions were significantly different in the fronto-central regions between imagery and perception trials. The modulation effects were further supported by the moderate negative correlations between (1) the attenuation of pain ratings of the recalled nociceptive images and (2) the amplitudes of attention shifting (P2) and imagery-related (P300 and N400) components. The moderate correlations between the pain ratings and scores on the Stroop Test suggest that response inhibition may play a role in focused attention and hence the modulation process.

### Orienting Attention and Nociceptive Perception

Participants tended to report lower pain NRS on imagery trials than on perception trials. However, the design of this study does not allow for definite conclusions about the positive effects of orienting attention for down-regulating pain perception. First, there was a potential exposure bias in the experimental design: Participants were exposed to a higher number of nociceptive stimulations during perception trials (first and second stimuli) than during imagery trials (just one stimulus). Second, average reduction in pain intensity in the imagery trials across all stimulation levels was −0.33 (average normalized pain NRS), which is rather small on an 11-point scale.

Third, the perception of the nociceptive stimulations could be confounded by repeated exposure throughout the trials. The mean pain NRS for level 5 stimulation was 3.97 (SD = 1.81) in the perception trials. In other words, the majority of participants gave ratings between 2.16 and 5.78 (1 SD = 64%). This was substantially lower than the calibrated pain NRS for the level 5 stimulations, which was 7 out of the 11-point NRS. It is plausible that the repeated exposure made the participants habituate to the stimulations, lowering their sensitivity and responsiveness. Hence the effectiveness of orienting attention on down-regulating pain perception is inconclusive.

### Early ERP Components in Orienting Attention

The nociceptive stimulation used in this study was very short–50 ms. Participants would need to perceive and register the sensation immediately after the presentation of the stimulation. Such processes appear to begin as early as the first 100 ms after the stimulus. The between-condition differences were mainly that the central SP1/2 (CN70–100) and SP2/3 (CTN100–180) were less negative in the imagery trials. These results may not readily compare with those in Dowman’s study [27], [29] because that study presented the auditory cue 1 s before the nociceptive stimulus. In contrast, the present study used low- versus high-pitched auditory cues presented at the same time as the brief nociceptive stimulation. The high-pitched auditory signal prompted participants not to appraise the nociceptive sensation, but to generate and mentally rehearse a learnt sub-nociceptive stimulus.

The simultaneous presentation of the auditory cue with the brief nociceptive stimulation could contaminate the SP(1/2), SP(2/3), and P2 (somatosensory-related) effects in this study. However, there are three observations that suggest that interference (if any) would be insignificant. First, the P1-N1-P2 complex (elicited from 50 to 200 ms), which is commonly associated with auditory stimuli, was found to have amplitude around 5 µV [43], [44], which was smaller than the somatosensory-related potentials obtained for the SP(1/2) (mean  = 11.38, SD = 2.84 µV), SP(2/3) (mean  = 10.82, SD = 3.4 µV), and P2 (mean  = 11.38, SD = 2.84 µV). Second, post-hoc independent component analysis using CURRY 6.0 revealed that the auditory-related P2 decomposed from the P1-N1-P2 complex (peaks at 196 ms) did not significantly overlap with the somatosensory-related P2 (peaks at 325 ms). Third, the differences in latency previously found between lower-pitched sounds (250 Hz) and higher-pitched sounds (4,000 Hz) [45] were not observed in the between-condition comparisons. These points indicate that any interference due to elicitation of the high/low frequency auditory cue (500 and 1,500 Hz) with the SP1/2, SP2/3, and particularly P2 would not be significant. These factors support the notion that our results can be compared to Dowman’s.

The less negative central SP1/2 and SP2/3 in the imagery trials could signify participants’ re-orientation of attention to the painful bodily sites from other attributes, i.e., visual cues [27]. Further studies by Dowman [29] suggested that the reduced amplitude of the early components were related to the cross-modal orienting of attention. Nevertheless, it is inevitable that the simultaneous presentation of the auditory cue and nociceptive stimulus (both were 50 ms) would cast a high cognitive demand on the participants. It is likely that the demand was an increase in attention load split between the two stimuli. The less negative centrally distributed SP1/2 and SP2/3 would reflect this process, which is perhaps different from that of Dowman’s study [29].

The next component revealed was a fronto-central P2 that was more positive during imagery trials. The result here is consistent with that reported in a previous study on focused attention, which associated P2 with bringing spatial attention to nociceptive stimulus [29]. Studies on response inhibition offer further insights into the functionality of P2. The fronto-central distribution of the P2 was found to be similar to the waves elicited in infrequent and deviant no-go trials that require participants to inhibit responses [5], [6]. There have been similar findings in studies that involved other senses: visual, auditory [17], [18], [20], and somatosensory [5], [6], [27]. The more positive-going P2 has often been associated with orienting attention toward infrequent target stimuli, withholding actions, and subsequently orienting attention away from the target stimulus [5], [6], [17], [18]. In other words, it reflects an inhibitory effect on the target stimulus. The frontally distributed P2 revealed in imagery trials of our study suggests plausible processes through which participants intentionally draw attention away from the nociceptive image elicited by the external stimulus. This would inhibit them from mentally rehearsing the nociceptive image further.

The behavioral results further support this idea. Both the amplitudes of P2 and scores on the Stroop Test correlated with the normalized pain NRS. Since the Stroop Test involves resolving two simultaneous stimulus conflicts by orienting attention to one attribute, this implies that the mental process reflected by P2 may signify orienting one’s attention from nociceptive stimulus to internally generate sub-nociceptive image. Previous research has found that the Stroop relates to monitory conflicts mediated by the anterior cingulate gyrus [46], [47]. Stuss and colleagues [39] further explained that monitory conflict–as a part of executive functioning–relies on attending to rules governing specific processing and behavior, such as the rule of naming colors in the Stroop Test. These offer convergent evidence on the process of conflict monitoring and resolution involved in the early part of the imagery trials after the presentation of the nociceptive stimuli [3], [22].

After the P2, there was a more positive P3 distributed over centro-parietal (CPz) sites. The temporal and topographical characteristics of P3 suggest that it possibly is a P3b component, which has been found to be associated with evaluation and categorization of sensory stimuli involving access to long-term memory [28], [30], [48], [49]. Previous studies showed that the P3b was particularly prominent when participants saw a rare target stimulus, regardless of its sensory modality [21], [50]–[52]. Other studies reported that it reflected evaluation subsequent to attending (which elicited a P2) to the stimulus involving working memory [48], [49]. The P3b revealed in this study suggested that participants were evaluating and categorizing the somatosensory information being focused on. The P3 elicited on imagery trials was more positive-going than on perception trials. This probably was due to the evaluation and categorization of the brief nociceptive stimulation required to generate the corresponding sub-nociceptive image from long-term memory for mental rehearsal. In contrast, the perception trials would only require maintaining and rehearsing the sensation felt. Nevertheless, it is not clear whether those processes were targeted at the incoming nociceptive stimuli or the sub-nociceptive images generated from within.

### Later Components for Imagery of Sub-nociceptive Sensation

The imagery trials elicited less negative N400 than the perception trials over the fronto-central areas. This late negative component could reflect the process of retrieving images the participants were told to retrieve from their memory. In this study those images were sub-components (e.g., location) of the pain sensation learned in the training before the experiment [53], [54]. Less negative N400 has been found to be associated with access to memory when generating and maintaining somatosensory [33] and visual images [31], [32]. This is consistent with our finding that imagery trials required participants to retrieve pre-learned sub-nociceptive images for rehearsing, whereas perception trials did not.

In addition, the N400 voltages elicited at many frontal to parietal sites correlated moderately with the normalized NRS ratings (levels 1 and 5) of the recalled nociceptive images. These frontal and central waves likely represent the maintenance of the self-generated sub-nociceptive images. The significant correlations at the two extreme intensity levels might be due to the fact that participants could more distinctly learn and recall them.

Nevertheless, the N400 has previously been found to be related to semantic processing, such as in reading [55], [56] and in processing non-linguistic stimuli, such as pictures [57]. The significant findings in the N400 could have been confounded if participants were doing semantic processing when they rehearsed the sub-nociceptive images. But the experimental design and results do not seem to support this speculation. All participants received training on recognizing and pairing the nociceptive and sub-nociceptive stimuli based on individualized voltage intensity and pain thresholds. In imagery trials, participants had to generate and rehearse a sub-nociceptive image corresponding to the brief nociceptive stimulus felt earlier. In perception trials, participants maintained a nociceptive image that was equivalent to what had been felt. These processes do not seem to involve semantic processing.

However, it is plausible that the N400 found in this study reflected some kind of knowledge integration, particularly when sub-nociceptive images were generated after the shift of attention from the incoming nociceptive stimuli. Emerging theories suggest that N400 reflects knowledge integration [55], [58]. Recent studies have suggested that N400 might reflect integrating knowledge when the meaning of the incoming information does not fit with their existing knowledge [55], [59]. Future studies are needed to test this using a somatosensory modality.

Although the P600 has been found to represent various kinds of higher-level functions (such as reasoning [31]), this LPC could be the extension of the earlier P300 component–particularly because the two showed up in the same posterior locations. Legrain and colleagues [28] found that the P600 (called the P3b due to its parietal distribution) was elicited when people were detecting infrequent deviants of somatosensory stimuli. This LPC was related to people retaining images in working memory or further sensory manipulation [60], [61], [62]. The centro-parietal topography of the LPC in this study was consistent with that revealed in Chow and colleagues’ study on imagery of vibrotactile sensation [33]. The only difference is that the P600 was more negative-going, whereas ours was more positive-going. The more negative-going LPC found by Chow and colleagues was elicited by rehearsal of the same vibrotactile images, the design of which was similar to the perceptual trials of this study. The positive-going LPC is probably related to retrieval of sub-nociceptive images after evaluation of the nociceptive stimulus (related to the centro-parietal P3). Our finding further supports the speculation that the LPC revealed is likely an extension of an earlier P300 elicited in the imagery trials. Its weak relationships with the normalized NRS ratings on the recalled nociceptive images suggest that this late process plays a less important role in modulating pain. This conclusion needs to be substantiated in future study.

### Conclusion

This study had participants orient attention from nociceptive stimuli and generate images of sub-nociceptive sensation to modulate perception of nociceptive images. The sequential focus-then-orient processing of somatosensory images incorporates (1) the focused-attention strategy, which places attention on the nociceptive stimulus felt, and (2) the distraction strategy, which switches attention to self-generated sub-nociceptive images and rehearses them. The electrophysiological results reveal that this two-step approach involved inhibitory processes of reorienting the attention away from nociceptive stimuli followed by generating, maintaining, and rehearsing the sub-nociceptive images in working memory.

These processes seemed to influence the evaluation of the prior nociceptive stimuli, resulting in modulation of the feeling of pain. Since emerging evidence suggests that chronic pain is associated with prefrontal lobe degeneration [3], the focus-then-orient attentional process developed in this study should be replicated on patients with chronic pain and/or frontal lobe dysfunction. The findings from that study could further substantiate the involvement of frontal lobe in pain modulation and shed light on the clinical application of this procedure to chronic pain patients.

The experimental task used in this study was relatively complex. The training prior to the experiment to ensure 80% accuracy did not control for the ways that participants generated and rehearsed the sub-nociceptive images. Potential variations among the participants in this top-down process could confound the results. Further studies may consider incorporating this aspect into the training and screening participants’ kinesthetic motor imagery ability to further reduce individual variation.

Future studies could use a subjective rating to reflect the vividness of image generation. Training participants to label the nociceptive stimuli (i.e., levels 1 to 5) might exert an anchoring effect on participants’ evaluation of the recalled nociceptive images by the end of the perception and imagery trials. It would be interesting to explore how these strategies could reduce variability among the participants. The concurrent presentation of the 50-ms auditory cue for differentiating perception and imagery processes could interfere with participants’ perception of the nociceptive stimuli (also 50 ms long). Future studies can use a different design to avoid the possible confounding effect imposed by the cue.

The nociceptive stimuli given to the participants were phasic (50 ms) rather than tonic, and the pain ratings assigned by the participants were based on recalled nociceptive images rather than current somatosensory stimulations. Future studies should examine the effect of focused attention on tonic and persistent pain and recruit participants with pathological pain. Another limitation is the use of post-3,000 ms nociceptive stimulations (50 ms) in the perception trials but sub-nociceptive stimulations (50 ms) in the imagery trials for the purpose of verification before participants gave subjective pain ratings. This would have heightened participants’ pain ratings and hence would have biased the results of the perception trials because participants had received more painful stimulation by that time.
